# The Modern Samaritan

**Published:** 1886-11-20

**Authors:** 


### THE MODERN SAMARITAN.

Perchance by many a rough road-side there lies
 One strip't of raiment, wounded and distressed,
Who now, with dying look, for man's compassion cries,
 And pitying breast.
If thine the lot to meet one so forlorn,
 With eager hands pour out thy wine and oil—
*Thy neighbour he:* oh, pass not by in scorn,
 God marks thy toil.

THE medical profession is not without curiosities in names. There are, for example Drs. Head, Hand, H(e)art, Legg, Foote, Tooth, and Body. A surgeon rejoices in the name of Cutmore, and a physician in the name of Coffin. We have also Dr. Death, and Dr. Liv(e)ing; a Dr. Easy and a Dr Payne.

